# Taphonomy of aquatic insects from the Crato Formation Lagerstätte (Aptian, Lower Cretaceous) under an actualistic look

**DOI:** 10.1371/journal.pone.0331656

**Published:** 2025-09-11

**Authors:** Arianny P. Storari, Frederico F. Salles, João Luiz Guedes da Fonseca, Antonio Álamo Feitosa Saraiva, Taissa Rodrigues

**Affiliations:** 1 Departamento de Ciências Biológicas, Centro de Ciências Humanas e Naturais, Universidade Federal do Espírito Santo, Vitória, Brazil; 2 Department of Entomology, Staatliches Museum für Naturkunde Stuttgart, Germany; 3 Museu de Entomologia, Departamento de Entomologia, Universidade Federal de Viçosa, Viçosa, Brazil; 4 Laboratório de Paleontologia, Centro de Ciências Biológicas e da Saúde, Universidade Regional do Cariri, Crato, Brazil; Universidade Federal do Pampa, BRAZIL

## Abstract

The Crato Formation (Aptian, Lower Cretaceous) is a fossiliferous deposit of global significance, representing a lacustrine palaeoenvironment which offers insights into aquatic insect taphonomy. Despite its importance, prior studies lacked an actualistic approach. Here, we analyze the preservation of mayflies (Ephemeroptera) and dragonflies (Odonata) from this formation using experimental taphonomy on 253 extant Ephemeroptera and 236 Odonata, alongside 306 fossil specimens. Disarticulation experiments showed that the thorax of modern mayfly larvae disarticulated first, yet Crato Hexagenitidae larvae retained intact thoraces, indicating minimal disturbance and autochthonous deposition. Fossil alate specimens rarely exhibited decay-related wing damage, aligning with short decay times. Dragonfly carcasses exhibited a characteristic leg posture in death, also preserved in Crato fossils, further suggesting minimal transport. Additionally, fossil dragonflies retained labial masks, the first structure to disarticulate experimentally, consistent with parautochthonous assemblages. Mayfly larvae exposed to low salinity during experiments exhibited excessive defecation before death, hinting at possible low salinity conditions in the Crato palaeoenvironment, though preservational challenges obscure confirmation. During experimentation, we also noticed that all carcasses immediately floated under hypersaline conditions, while carcasses immersed in non-hypersaline conditions went through slower decomposition. Thus, we can safely propose with experimental data that microbial biofilms on the surface of the water were acting during carcass sinking in this deposit.

## Introduction

The Crato Formation holds some of the most interesting fossiliferous deposits in the world [1]. It is especially important for palaeoentomology due to its Lower Cretaceous age, location, and the terrestrial origin of most of its fossils, since the majority of other important deposits for the study of insects are both of non-terrestrial origin and occur in the Northern Hemisphere [2,3]. Nevertheless, since it mostly represents a lacustrine palaeoenvironment [4], it is particularly important for the study of its aquatic insects and their interaction with this past environment. The most prominent aquatic insects occurring in the Crato Formation are Ephemeroptera and Odonata, which present an outstanding abundance, diversity, and exceptionally preserved specimens [5].

Martins-Neto and Gallego [6] postulated an interesting concept, namely thanatoethology, based on the observation of fossil insects from the Crato Formation, especially Orthoptera. Thanatoethology refers to the identification, interpretation, and study of a specific behaviour performed by an organism moments before its definitive death, i.e., before the beginning of the fossilization process. This behaviour expresses agony, often due to asphyxia in land, air, or water, and can be expressed in fossils afterwards, providing important clues to unravel taphonomic processes acting prior to the mineralisation of a carcass. However, most studies on the fossil insects of this unit focused on taxonomy, including the aquatic groups addressed here, Ephemeroptera and Odonata [7,8,9,10,11,12,13,14,15,16,17,18,3,19,20,21,22,23].

Recently, several investigations that analyzed the general insect taphonomy of that unit have been published [5,24,25,26,27,28,29,30,4,21,31,32,33,23,34] and show that arthropod assemblages can provide important clues about the nature of the palaeoenvironment. Freshwater taxa are especially taphonomically informative, as they lived and died in the depositional site (or near it, in the case of terrestrial adults). For instance, fossil mayflies from the Crato Formation are ideal for quantitative analyses because their abundance (in the hundreds, if not thousands, of excavated individuals) allows good sampling [21]. Among them, the Hexagenitidae (Ephemeroptera) is the most common family of arthropods found in that deposit (specifically *Protoligoneuria limai* assemblages), with their larvae representing 85% of the total number of insect specimens in a controlled sampling of the C6 limestone sequence [21]. The Odonata are also well-represented [33]. Therefore, the study of the taphonomy of these two groups can provide insights into some of the phases of the Crato palaeoenvironment.

In this work, we focus on the biostratinomy of the aforementioned aquatic groups, in order to better understand the processes that led to this important fossiliferous deposit. The disarticulation and transport, for instance, tend to be two closely related parameters, as the first often occurs due to the latter, although disarticulation can occur naturally with no disturbance [35,6]. Disarticulation is also associated with a prolonged period between the death of an organism and its burial, so if burial occurs before complete necrolysis of the soft tissue, a carcass could be preserved practically intact and articulated [36].

A branch of taphonomy, traditionally underexplored for the Crato Formation fauna, is the actualistic taphonomy, also investigated in this work. It compares the fossil record with experiments on the preservation and decomposition of extant animal and plant carcasses and can provide important palaeoenvironmental data [37]. There are only a few studies that explored experimental data on modern fauna as a proxy for studying the Crato fossils. For arthropods, spider leg flexion was used by Downen et al. [38] for inferring salinity in the palaeolake, and Iniesto et al. [30] studied the role of microbial mats in the decay of the larvae of an extant moth and mealworm species. Recently, Dias et al. [39] also compared microscopic signatures supposedly left by microbial mats in mayfly larvae fossils with signatures left by modern microbial mats in salt pans. Arthropods are good candidates for actualistic analyses because many experiments can be done under controlled laboratory environments, and most fossil groups have modern equivalents. For instance, the overall body shape of larval stages of Hexagenitidae mayflies is similar to representatives of Tridentiseta (sensu [40]) or Pisciforma (sensu [5,41,42]), which usually have streamlined bodies [43]. Among them, the extant family Baetidae is widely distributed on all continents, except Antarctica [44,45] and, since it is the only Tridentiseta clade occurring in Brazil [46], it is the appropriate group for taphono-comparative actualistic studies in the country. Since Hexagenitidae (*Protoligoneuria limai* assemblages) is by far the most abundant arthropod group of the Crato Formation and the only well-represented one with available controlled collection data, it was our focus group during this study. However, to broaden our understanding of the aquatic biota and to compare the results among the most prominent aquatic insect groups occurring in that unit, we also assessed the taphonomical patterns of the Odonata and examined data from extant larval individuals. Thus, we performed experiments to determine the nature of the taphonomic processes responsible for the biostratinomic preservation of larval and winged mayflies and larval dragonflies of the Crato Formation.

## Materials and methods

### Extant specimen collection

**Fig 1:**
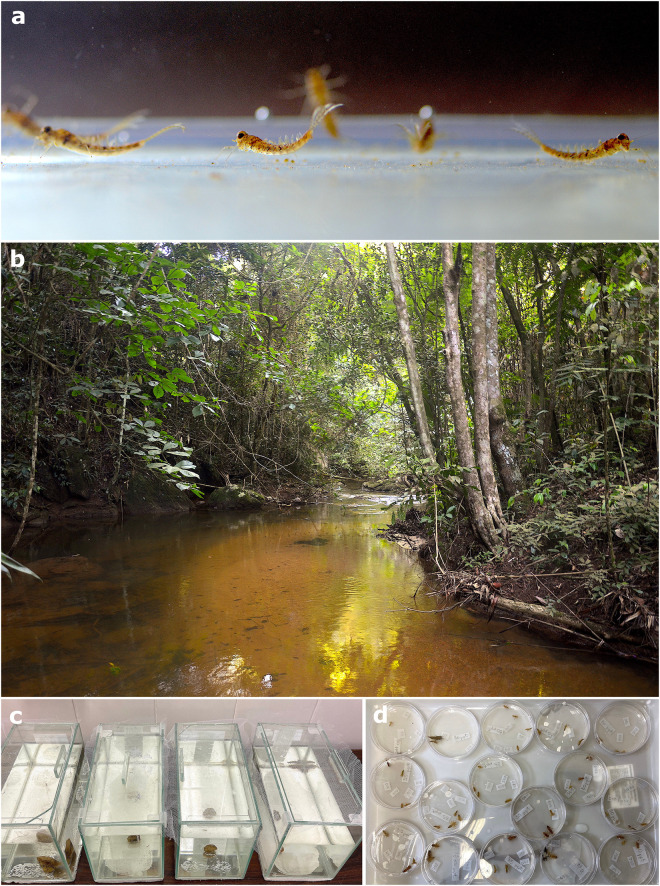
Collection site and setup of actualistic experiments. a) Living *Callibaetis capixaba* larvae (Ephemeroptera: Baetidae) during the experiments. b) One of the sampling points in the Atlantic forest of Nova Lombardia, Santa Teresa municipality, Espírito Santo, Brazil. The preferred habitat of *C. capixaba* larvae was sandbanks and overall sandy substrate of semi-lotic streams. c) Glass aquariums where experiments were conducted with the sampled specimens: two aquariums for salinity tests and two for temperature tests. d) Petri dishes with dragonfly carcasses during disarticulation tests.

**Fig 2:**
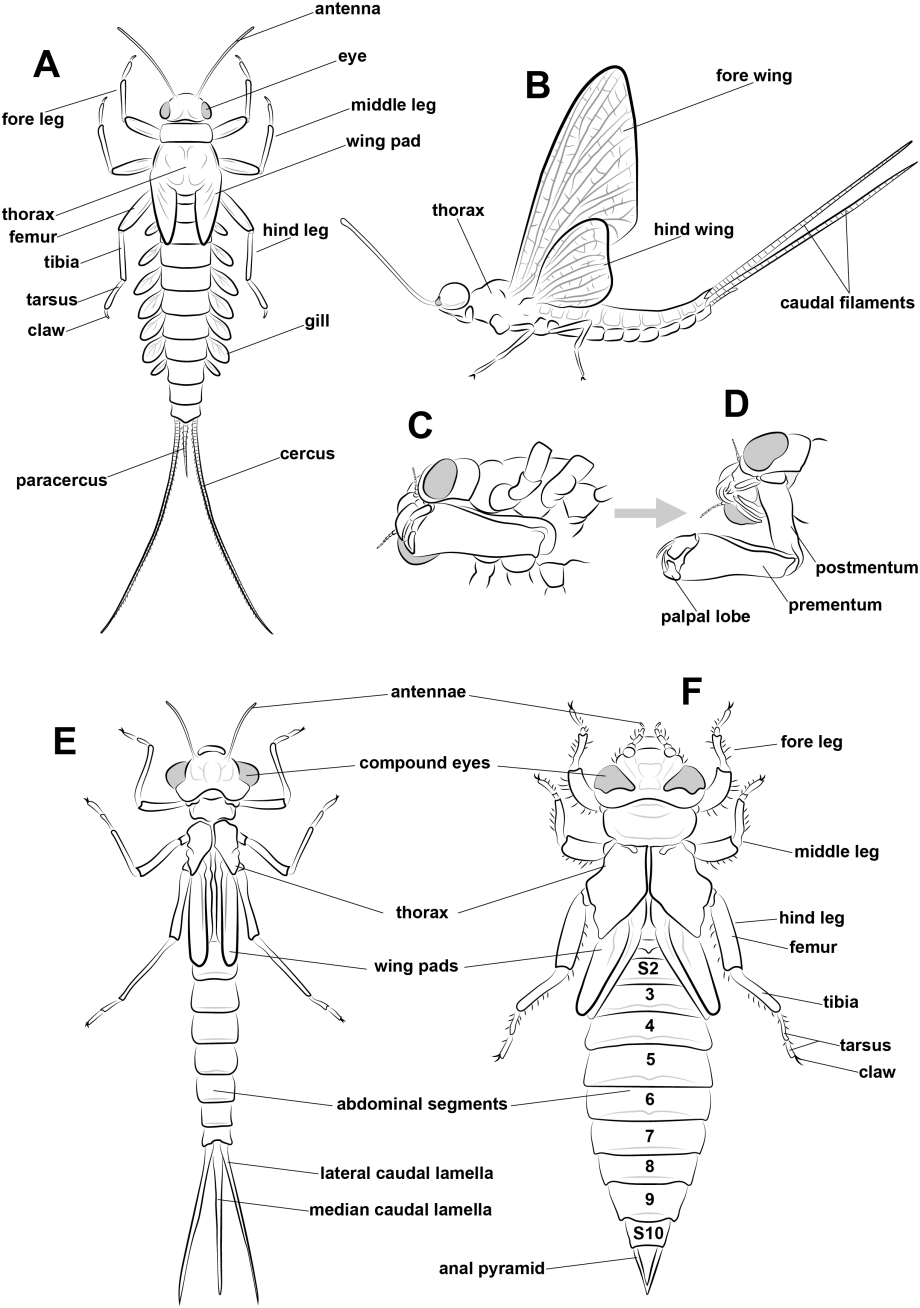
Schematic anatomic drawing of larva and adult Ephemeroptera and larval Odonata. a) General mayfly larva anatomy of pisciform body type of *Callibaetis capixaba* . b) General anatomy of adult mayfly. c) Ventral view of head anatomy of dragonfly larva displaying its labial mask at rest d) Ventral view of head anatomy of dragonfly larva displaying its labial mask extended (c,d adapted from [47]). e) General Odonata larval anatomy displaying a Zygoptera body plan. f) Anisoptera bodyplan (e,f adapted from [48,49]). All drawings made by Richard Buchmann (Vitória, Brazil).

To perform the experiments with extant specimens, a total of 253 *Callibaetis capixaba* [45] larvae (Ephemeroptera: Baetidae), and 236 Odonata larvae (vast majority of them Gomphidae - Anisoptera, but also the Anisoptera families Aeshnidae and Libellulidae, and the Zygoptera families Calopterygidae and Coenagrionidae) were collected in Santa Teresa municipality, Espírito Santo (Brazil), in the locations Nova Lombardia (S 19° 52′ 31.6′′and W 40° 31′ 49.1′′) and Augusto Ruschi Biological Reserve (S 19° 53′ 20.6′′and W 40° 32′ 41.5′′). All necessary permits were obtained for the described study, which complied with all relevant Brazilian regulations under sampling authorization number SISBIO 81584-1 (three sampling trips in the years of 2021 and 2022). Besides its morphological similarities with Hexagenitidae, we chose *C. capixaba* among other baetids because it presents a reasonable size for macroscopic observations (ca. 10 mm), and can be easily collected in sandbanks, its preferred habitat (Fig 1). The odonatan groups collected were available while sampling in the preferred habitat of *C. capixaba* and share similarities in body plan with Odonata taxa occurring in the Crato Formation. The general anatomy of the aquatic insects used in this study is displayed in Fig 2.

Collections were made using sieves and nets with mesh openings of 0.5 and 1.0 mm. The taxonomic identifications were based on Cruz et al. [50]. Salinity, pH, and temperature conditions in the field were measured to establish values for the control groups using a Yieryi Portable refractometer, disposable PRODAC pH tests, and a Boyu glass thermometer. The specimens were placed in individual plastic containers for transport, following the protocol of Boldrini and Cruz [51], and reared in glass aquariums with filtered water from the collection site (Fig 1). After the end of the experiments, the specimens were deposited at the Museum of Entomology of the Federal University of Viçosa (Viçosa, Minas Gerais, Brazil) in a collective batch (Storari sampling, Santa Teresa, 2021).

### Experiments

**Fig 3:**
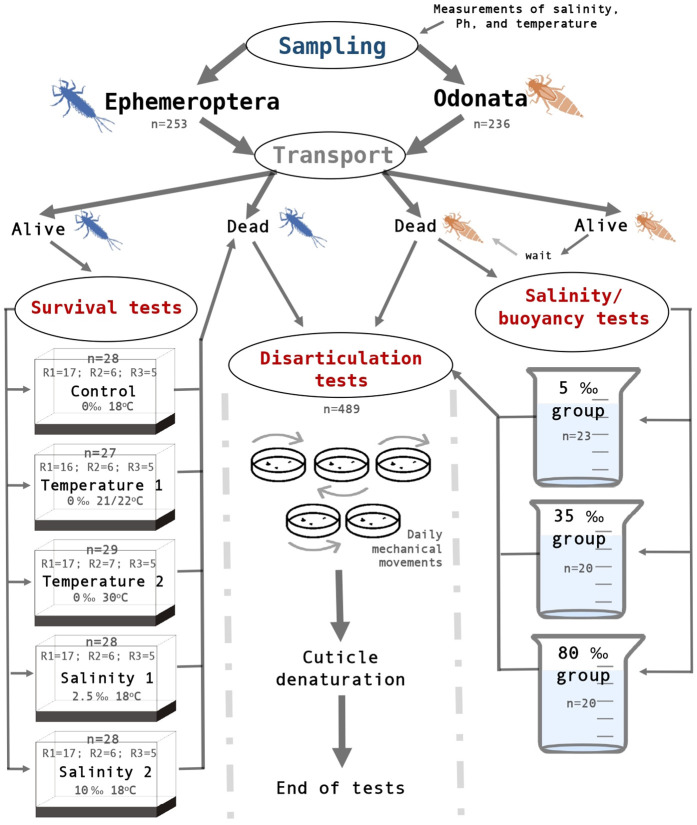
Schematic drawing of the experiments. Simplified infographic of our experiments’ steps, from sampling to the end of tests. ‘n’ is the total sample number (from the three replicates together, which represents three sampling trips). R = replicate.

Experiments were done over six months and included three replicates (R1, R2, and R3). We performed the main experiments with mayflies (including survival rates and disarticulation tests) and additional experiments with dragonflies (tests on disarticulation and flotation under different salinity rates) (see Fig 3 for simplified schematization of experiments; and S1 and S2 Tables for results with extant Ephemeroptera and Odonata specimens, respectively).

Ephemeroptera larvae were split into groups and then allocated in aquariums (see section *Survival rate tests* below for specifications on conditions of aquariums and groups). Odonatans were also allocated in aquariums with water conditions of 0‰, 5.5 pH, and 18°C. The specimens were reared until their natural death. After that, some of them were placed in the salinity/flotation test, and the remaining in the disarticulation test (Fig 3).

We statistically tested the differences between different salinity and temperature rates using log likelihood ratio tests of independence (G-test) using the package DescTools (Signorell, 2025[52]), and figures were made using the package ggplot2 (Wickham, 2016), both in R 4.4.1[53,54].

During and after the experiments, the specimens were examined using a Leica MZ7.5 binocular stereomicroscope (0.63–5.0x objective lens, 10x eyepieces), and photographs were taken with a Nikon D7000 DSLR camera. When possible, serial photographs with different focal planes were taken, and the photo stacks were posteriorly processed with Helicon Focus Pro 6.4.1.

#### Survival rate tests.

This test was performed with alive specimens in the lab to evaluate whether mayflies would present different survival rates and different trends of post-mortem attitudes under different abiotic conditions.

An aquarium was assigned to the control group (with 28 *C. capixaba* larvae in total for the three replicates – R1 = 17; R2 = 6; R3 = 5), in which controlled salinity (‰), pH, and temperature (°C) conditions were established based on those of the collection site (water conditions of 0‰; 5.5 pH; and 18°C) (Fig 3).

Four aquariums were destined for the test groups: two for salinity tests – one with 2.5‰, 5.5 pH, 18°C, with 28 *C. capixaba* larvae at total for the three replicates (R1 = 17, R2 = 6, R3 = 5), and the other with 10‰, 5.5 pH, 18°C, with 28 *C. capixaba* larvae at total for the three replicates (R1 = 17, R2 = 6, R3 = 5); and two for temperature tests – one with 21–22°C, 0‰, 5.5 pH, with 27 *C. capixaba* larvae at total for the three replicates (R1 = 16, R2 = 6, R3 = 5), and the other with 30°C, 0‰, 5.5 pH, with 29 *C. capixaba* larvae at total for the three replicates (R1 = 17, R2 = 7, R3 = 5) (Fig 3). Values were chosen based on the control conditions of the sampling area and the biological limits that mayflies are known to inhabit [55]. The data was tested for differences between categories with a Kruskal-Wallis rank sum test after checking for normality with a Shapiro-Wilk test, and with a Levene’s test for homogeneity of variance, using the basic statistics R package [53]. A post hoc Dunn’s multiple comparison test with Bonferroni correction was performed to identify significant pairwise differences using the package DescTools (Signorell, 2025) in R 4.4.1 [53].

For allocation from the transport containers to the aquariums, a pipette with a thick tip was carefully used to handle specimens one by one (alive or carcasses). No substrate was placed in the aquariums as it would make observation of the small larvae difficult, and the aquariums were covered with a protective net on the top (Fig 1). Dead leaves whose tissues had already been destroyed by bacteria were added to feed the larvae (Nikita Kluge, personal communication). The first batch of aquarium water was collected at the sampling site. The water was renewed with filtered water every 2–3 days to prevent bacteria and algae from proliferating [56].

In the salinity tests (two experimental groups), the larvae were subjected to an increase in the water’s salinity, with dilution of different concentrations of saline solutions using Instant Ocean Sea Salt, a mixture commonly used in saltwater aquariums, and measured using the refractometer. The laboratory’s overall temperature was controlled daily with an air conditioner to keep the aquariums’ water temperature at 18°C, while the two aquariums with increased temperature each had a Roxin aquarium heater (25W).

All larvae were reared until their death or until they reached the winged stage (i.e., subimagoes). The aquariums were checked daily at the same time to record the number of deaths and/or emerging specimens. In the latter case, we simply waited till the specimen died naturally, attached to the aquarium net or drowned after falling into the water. After the death and/or moulting of the larvae, we counted the number of individuals that reached their final winged stages (subimago or imago) in each experiment. We registered the post-mortem positions of all larvae and alate forms in the aquariums. Unfortunately, four specimens that escaped through the aquarium’s upper net holes could not be recovered for counting. Each replicate ended once all the larvae died or emerged, and after all of them were accounted for in the disarticulation tests (see below), a new field collection was performed.

#### Salinity versus flotation tests.

This test aimed to evaluate how dragonfly carcasses behaved with the increase of salinity in regard to flotation time and leg flexure, similar to the test of Downen et al. [57,38] with spiders. Salinity values were, however, modified from these authors based on standard freshwater, seawater, and hypersaline water values [58].

Sixty-three odonatan larval specimens were selected immediately after their death for immersion in saline water with different concentrations (freshwater – 0.5‰, 5.5 pH, and 18°C, with 23 specimens; saltwater – 35‰, 5.5 pH, and 18°C, with 20 specimens; and hypersaline water – 80‰, 5.5 pH, and 18°C, with 20 specimens) to assess carcass flotation [59,38]. The larvae from each group were placed in the same glass beaker, and the saline solution was poured over them to break the surface tension. Each vial was filled with the solution until full and then covered with plastic film. Saline and hypersaline solutions were created using Instant Ocean Sea Salt. The glasses were kept completely still and were photographed and observed every day to check if specimens started floating or changed the angle of their legs. After the end of tests, the carcasses were also evaluated under disarticulation tests (see below).

#### Disarticulation tests.

This test was performed to evaluate disarticulation trends of different body parts of Ephemeroptera and Odonata.

All the collected specimens were quantified regarding their disarticulation rates. Besides the mayfly and dragonfly larvae, we also examined 15 winged Ephemeroptera, including 10 *C. capixaba* and five representatives of Leptophlebiidae that were collected by accident. We chose to also analyze them since their adults have the same overall morphotype as *C. capixaba*, though their larvae differ.

After the death of the *C. capixaba* and of the odonatan larvae, we assessed disarticulation steps by carefully leaving the specimens to decompose in Petri dishes filled with water from the control group aquariums under different time slots. The specimens that died during transport from the sampling place to the lab, and thus did not participate in the survival tests, were also allocated to the Petri dishes for the disarticulation observations. Specimens in the water were subjected to controlled, gentle movement by hand, for one minute, to evaluate the most sensitive parts to disarticulate under low mechanical movements. Every second day, we took notes on new elements disarticulating at hand scale (since specimens were only analyzed individually and in detail with a binocular stereomicroscope after the end of tests). Once all the analyzed specimens (or the majority of specimens in an Petri dish) started to show signs of cuticle denaturation (i.e., cuticle presented as faded and transparent), we took notes on the state of the carcass with a binocular stereomicroscope (i.e., which limbs were disarticulated at that moment), and preserved them in plastic microtubes with 70% alcohol at room temperature, as vouchers.

### Fossil material

We analyzed a total of 306 fossil specimens: 230 Hexagenitidae larvae, 31 Hexagenitidae alates (subimagoes and imagos), and 45 Odonata larvae (28 Proterogomphidae and 17 Nothomacromiidae). To prevent bias towards well-preserved individuals only, most of the Hexagenitidae larvae of the Crato Formation we analyzed were from the controlled collection at the Mine Antônio Finelon (S 07^o^ 07′ 22,5′′and W 39^o^ 42′ 01′′), Nova Olinda, Ceará State (225 specimens). Since few alate Hexagenitidae specimens were recovered in that excavation, we also included literature data in our study [7,8,11,13,39,18,60,61,29] and direct observation of specimens of the Palaeontological collection of the Universidade Regional do Cariri – LPU URCA (Crato, Brazil), the Palaeontological collection of the Museu de Paleontologia Plácido Cidade Nuvens – MPSC (Santana do Cariri, Brazil), and the Scientific Palaeontological Collection of the Institute of Geosciences of the Universidade de São Paulo – IG USP – GP (São Paulo, Brazil) (S3 and S4 Tables).

To carry out biostratinomic analyses, the following taphonomic signatures were assessed and quantified: ontogeny (larva or adult and their body length), attitude (fossil preserved dorsally, ventrally, or laterally), and disarticulation. Disarticulation was measured qualitatively and quantitatively, accounting for the total body elements missing from each of the specimens (e.g., specimen X is missing 1 head + 2 legs + 3 gills).

## Results

### Actualistic data of mayflies

**Fig 4:**
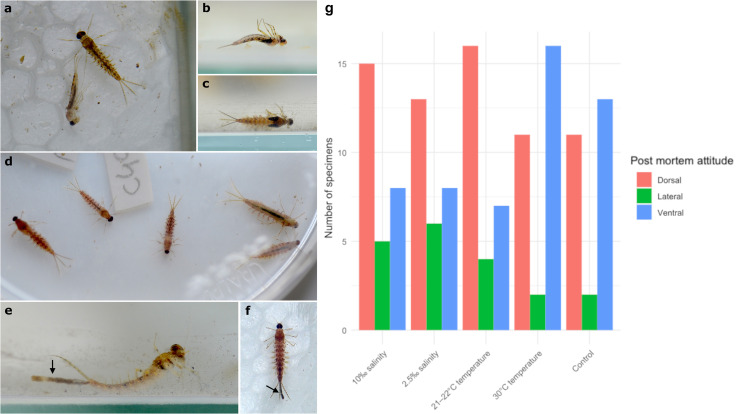
*Callibaetis capixaba* larvae during survival tests. a) Two *Callibaetis capixaba* carcasses, in dorsal view (right) and right lateral view (left). b) *C. capixaba* carcass in ventral view. c) *C. capixaba* carcass in dorsal view. d) Five larval carcasses of *C. capixaba* that died under high temperature, showing a reddish coloration. e,f) *C. capixaba* larvae allocated at lower salinity levels and defecating in unusual amounts; ‘f’ is already dead. Black arrows point to the excessive excrement eliminated by the larvae. g) Graph with number of specimens recovered in different postmortem attitudes (dorsal, lateral, or ventral view) on y-axis, for each group of survival tests on x-axis (control, 2.5‰ salinity, 10‰ salinity, 21–22°C temperature, and 30°C temperature).

We observed that the usual *postmortem* position of the *C. capixaba* larvae, in all survival rate test groups, was either in dorsal view (i.e., ventral decubitus, 47% of specimens) or in ventral view (i.e., dorsal decubitus, 31% of specimens) (Fig 4a–4d and 4g). When the larvae died in dorsal decubitus (ventral view), they remained in a characteristic position with their legs not flexed but extended laterally near the body, with the forelegs extended forward (Fig 4a and 4d). The legs were flexed over the body when in dorsal view (Fig 4b). Individuals in lateral view/decubitus after death (22% of specimens) were observed being disturbed by the activity of other larvae and/or minor currents in the aquarium (Fig 4a, 4c and 4g). However, the G-test results showed no significant differences in *postmortem* position between the different salinity and temperature rates (df = 12, p-value = 0.312).

Fifteen specimens of the control group survived and reached the winged stage, combining the three replicates (R1 = 13, R2 = 1, R3 = 1). No specimens allocated in the experimental groups reached the winged stage, varying only in the time the larvae could survive before dying. All specimens allocated under a higher temperature (30°C) survived at most two days after allocation, and after death, their bodies displayed no natural curvature and had a reddish colouration (Fig 4d). The specimens allocated at 21–22°C survived longer, on average 5.96 days (ranging from 3 to 10 days), and no change in colour or posture occurred, differing from those in 30°C.

Specimens allocated in more brackish water (10‰) also died quickly, with most of them dying around one day after allocation in the aquarium (n = 25), while three specimens from the R1 survived for two days. Only three out of 28 specimens allocated at lower salinity levels (2.5‰) were alive for one week (all from R1), but the remaining 25 died within four days (average: 3.27). In the latter group, the larvae were defecating in unusual amounts long before death, always one or two days after allocation to the aquarium (Fig 4e and 4f; Table 1). The Kruskal-Wallis test was significant (chi-squared = 88.391, df = 4, p-value < 0.05). Pairwise differences were significant between the control group and the 10‰ salinity group (p.adj = 0.0000000001), the control group and the 30°C temperature group (p.adj = 0.00000003), between the 2.5‰ and 10‰ salinity groups (p.adj = 0.000001), and between the 21–22 and 30°C temperature groups (p.adj = 0.000000009). The remaining pairwise comparisons were not statistically significant.

**Table 1:** Survival rates of experimental groups. Number of days specimens survived at each group of survival tests (control, 2.5‰ salinity, 10‰ salinity, 21–22°C temperature, and 30°C temperature) and their average number of survival days per group.

Group	Number of days alive	Number of specimens	Average
control	1	6	8.28
control	3	2	
control	4	2	
control	5	1	
control	7	1	
control	11	12	
control	15	2	
control	19	2	
21−22°C	3	6	5.96
21−22°C	4	1	
21−22°C	6	11	
21−22°C	7	3	
21−22°C	8	3	
21−22°C	9	2	
21−22°C	10	1	
30°C	1	18	1.41
30°C	2	10	
30°C	3	1	
2.5‰	2	5	3.85
2.5‰	3	6	
2.5‰	4	11	
2.5‰	5	3	
2.5‰	7	3	
10‰	1	25	1.10
10‰	2	3	

**Fig 5:**
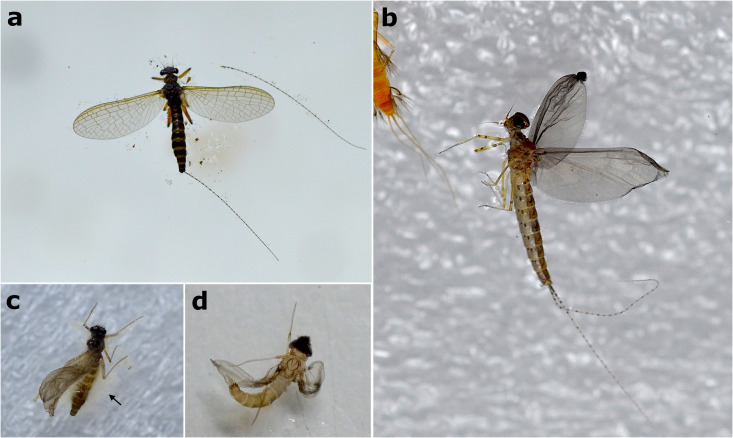
Winged mayflies during disarticulation tests. a) The most frequent postmortem position for winged mayflies; *Askola* (Leptophlebiidae) female imago floating on the water surface with wings open and spread out, in dorsal view. b) Winged male *Callibaetis capixaba* subimago that died while still attached to the protective net trying to moult to imago stage, floating on the water surface with wings adducted. c,d) Female and male *Askola* subimagoes, respectively, floating after days of decomposition, with their wings withered and abdomen shrunk, as well as whitish microbial proliferation on the abdomen in ‘c’ (black arrow).

Due to difficulties in handling and keeping emerging alate forms in a controlled environment, we only observed the natural death of seven winged individuals. The remaining eight alate specimens that emerged had to be euthanized in alcohol when about to escape. After that, the specimens were submerged in water for cleaning and then placed in the Petri dishes for subsequent disarticulation tests. From those seven specimens that died naturally, five emerged and then drowned, presenting their wings open and spread out, while two subimagoes died while attached to the aquarium protective net, without falling into the water, and presented their wings adducted (Fig 5a and 5b). Without any water disturbance, the winged carcasses did not sink to the bottom of the aquarium and went through decomposition without ever sinking. The soft-tissued abdomen began to decay immediately after death, and bloating, likely due to fermentation gases in the abdomen, facilitated buoyancy. On average, 15 days (ranging from 4 to 25 days) after allocation in the disarticulation tests, the carcasses had a shrunken abdomen and completely withered wings, then we ceased the disarticulation observations (Fig 5c and 5d; S1 Table).

**Fig 6:**
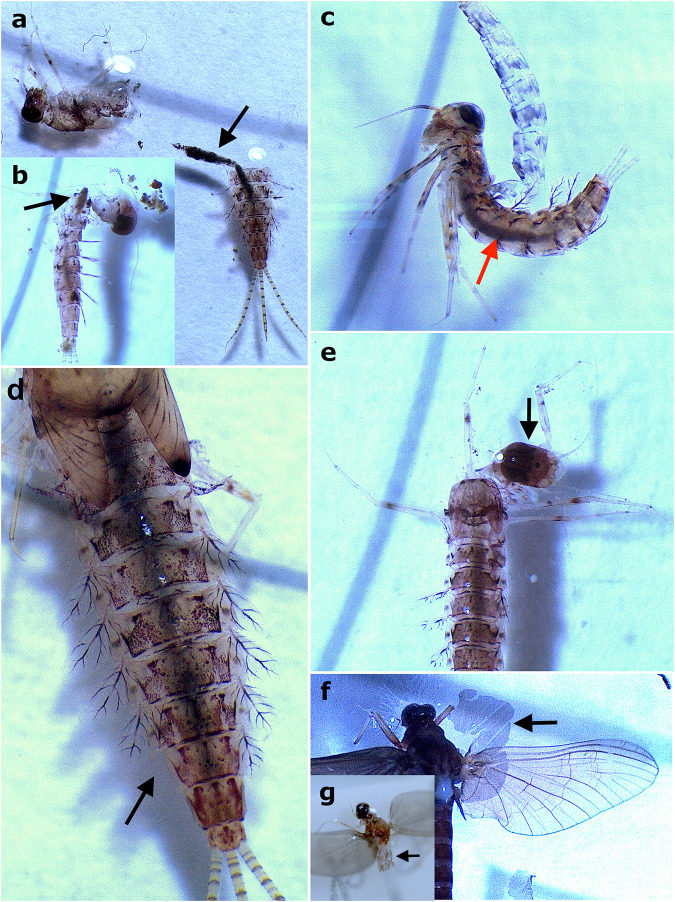
Larval and winged mayflies during disarticulation tests. a,b) *Callibaetis capixaba* carcasses with thorax disarticulated, in which darkening and detachment of the digestive tract are also evident. Digestive tracts indicated by black arrows. c) *C. capixaba* carcass with darkened digestive tract and part of caudal filaments disarticulated. Digestive tract indicated by red arrow. d) *C. capixaba* carcass with one final gill missing, indicated by black arrow. e) *C. capixaba* carcass with head disarticulated. Head indicated by black arrow. f) *Leptophlebiidae* carcass with subimaginal membrane of forewing detaching. Piece of membrane indicated by black arrow. g) *C. capixaba* male subimago with missing abdomen (except by first segments indicated by arrow).

Regarding the disarticulation observations, the darkening and detachment of the digestive tract were one of the first processes that occurred during larval decomposition (usually less than a day after death) (Figs 3d and 5a–5e) and possibly influenced the disarticulation of other corporal elements, such as the thorax. The latter was the most likely element to disarticulate first naturally: 26% of specimens had their thorax disarticulated, on average 3.5 days after their death (Figs 6a, 6b and 6h; 6). The disarticulation of the thorax occurred faster in situations of denaturation acceleration, such as in warmer waters, in which, in most cases, it disarticulated just one day after the death of the individual (even before allocation to Petri dishes, when the specimen was noticed dead in the daily check) (Fig 6h). Many specimens started to show early signs of cuticle denaturation when decomposing in water (when disarticulation tests ceased), with 108 specimens within 2–4 days only, with the average number of days for all the larval mayflies analyzed being 4 (see S1 Table). The redder and more transparent the cuticle was, the more decomposed the specimens were.

**Fig 7:**
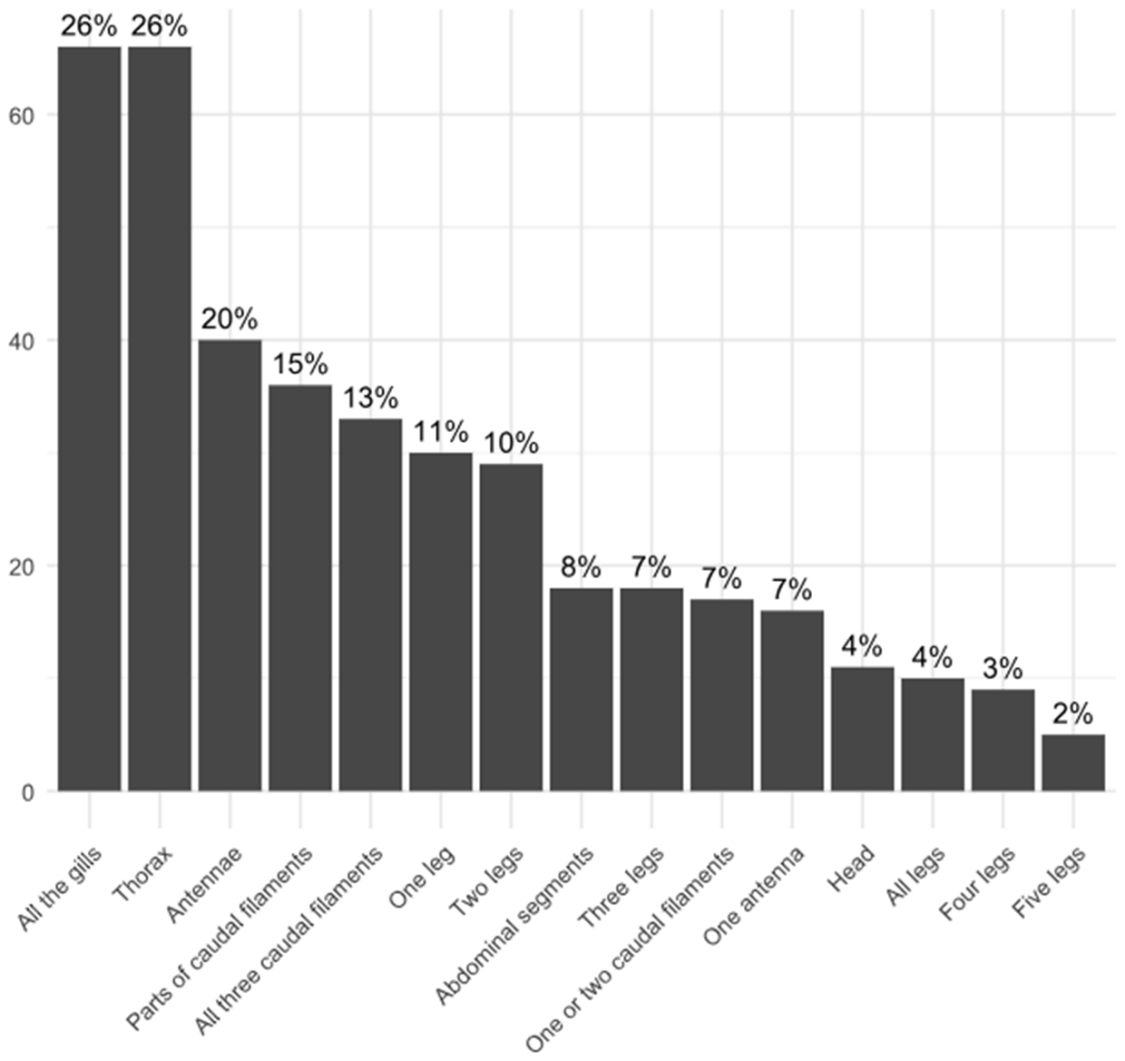
Disarticulation rates of larval mayflies. List of body parts disarticulated on x-axis, and the total number of specimens with this part disarticulated when observations ceased (see text) on y-axis, and what percentage this number represents over columns. Percentages are rounded up.

We observed that carcasses that went through natural disarticulation in the Petri dishes more frequently had the thorax as the first body part to disarticulate or at least weaken. In the larvae that died in the aquariums alongside several alive and active larvae (disturbing the carcasses before the specimen was noticed dead in the daily check), the legs, gills, caudal filaments, and antennae (most delicate body parts) were the first body parts to disarticulate. In the specimens that lost only some of their gills, the last ones were more prone to disarticulate first (Fig 6c and 6d). For complete percentages of disarticulated body parts, see Fig 7.

In alate forms, the first elements to disarticulate were the thorax and wings. The membrane of the subimagoes' wings also detached easily after the carcass decomposed (Fig 6f and 6g). The abandoned larval exuvia tended to disarticulate in the weak spots between thorax and head, and between thorax and abdomen (S1 Table).

### Actualistic data of dragonflies

**Fig 8:**
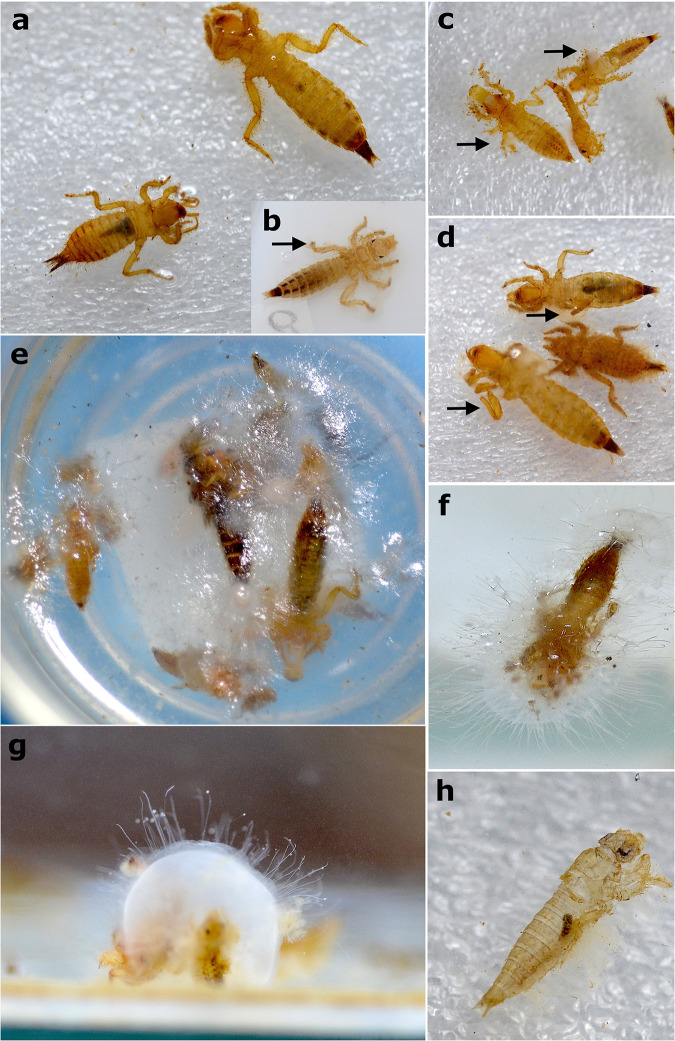
Dragonfly larvae during experiments. a) Gomphidae larvae stressed in ventral view and close to die, exhibiting the characteristic position with the hind legs distended laterally and the forelegs extended anteriorly. b,c,d) Gomphidae carcasses after minor disturbance. Black arrows point to the disarticulated hind legs (in different points: in ‘b’ tibia, ‘c’ tibia and femur, and ‘d’ coxa and femur. e,f) White microbial consortia covering floating carcasses. g) Carcasses at the bottom after descending from flotation due to the weight of microbial consortia. h) Carcass showing signs of chitin denaturation in which the cuticle starts to become transparent.

When dragonfly larvae were close to dying, they usually stayed in ventral view and without any movement. After dying either in dorsal or ventral view (more frequently in ventral; 64% of specimens, n = 151), they stayed in a characteristic position with the hind legs extended laterally and the forelegs extended close to the body (S2 Table). After disturbance, the extended hind legs disarticulated completely, either at the base of the femur or between the femur and tibia (Fig 2), likely because they were more exposed (Fig 8a–8d). All the larvae tended to float after dying, probably due to the gases released inside the abdomen by the decomposition. Dense white microbial consortia quickly appeared in 3–4 days (similarly to the mayflies but in smaller amounts in the latter – which were not quantitatively analyzed as for dragonflies), which seemly helped to keep the labium and legs articulated. This step was followed by the darkening and denaturation of the head*.* After the microbial chitinophagous consortia attacked, the denaturation accelerated, and after only one week, the animal was very disintegrated (signs of disintegration are visible when the cuticle starts to become transparent by the decomposition of chitin) (Fig 7e–7h), upon which tests were ended. The average time for disarticulation tests to end was 14 days (see S2 Table). We also recorded one case of cannibalism, possibly caused by stress, 15 days after the allocation of specimens in the aquariums.

**Fig 9:**
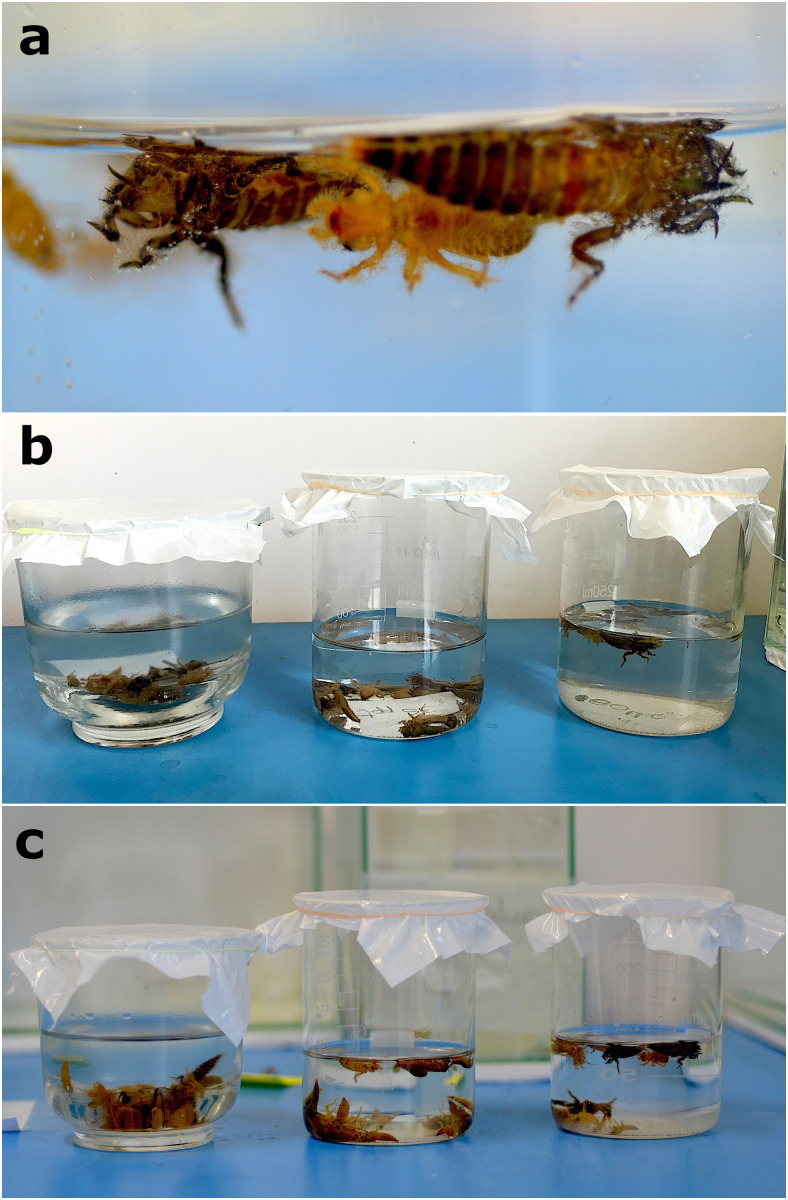
Dragonfly larvae during salinity tests. a) Close-up of floating carcasses during immersion tests in saline water. b) First day of the immersion tests with saline water; from left to right groups 0.5‰, 35‰, and 80‰ (in which the carcasses immediately floated). c) Second day of experimentation, in which carcasses of the 35‰ group came to float, while carcasses from the 80‰ group started to descend back to the bottom.

During the salinity versus flotation tests, we found that the angle of the legs of the carcasses was not affected by the increase in salinity (even using significantly higher values than [38]), but flotation was (G = 137.77, df = 4, p-value < 0.05). Under hypersaline conditions (80‰), all Odonata carcasses immediately floated, and in lower and seawater-like salinity (0.5‰ and 35‰), they all sank to the bottom of the glass after the saline solution was poured over them. The 20 carcasses allocated at 35‰ seawater salinity only started to float in the vials two days after immersion, while the 23 carcasses allocated at 0.5‰ salinity floated after three days (Fig 9).

**Fig 10:**
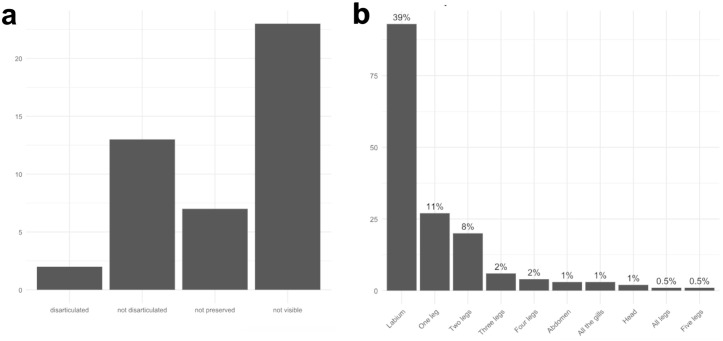
Rates of labial disarticulation of fossil dragonflies. a) Labial mask condition after experiments, total number of specimens on y-axis; b) Graph of main body parts disarticulated on x-axis, with total number of specimens with this part disarticulated y-axis, and what percentage this number represents over columns. Percentages are rounded up.

**Fig 11:**
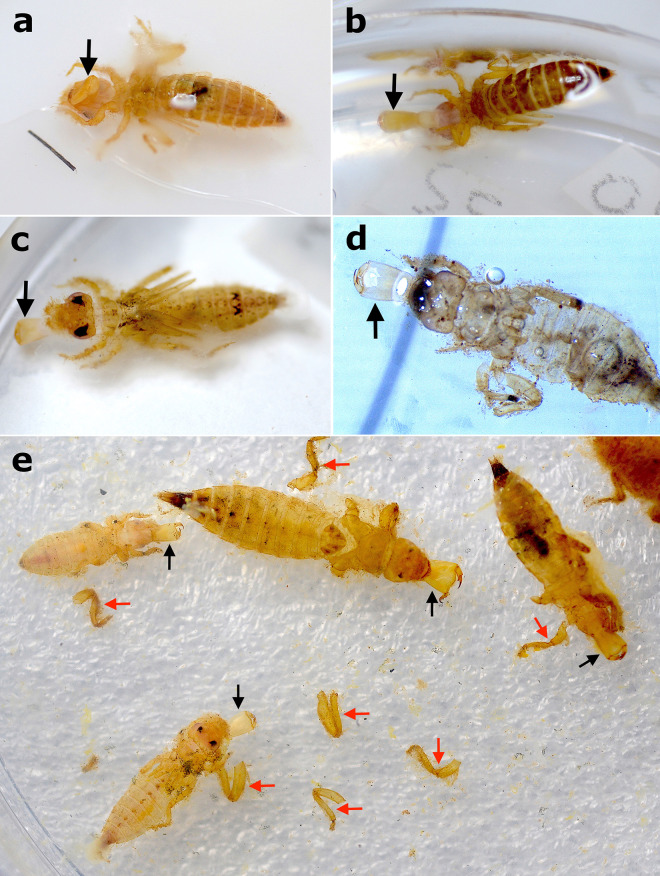
Gomphidae larvae during disarticulation tests. a) Larva in ventral attitude with labium in early process of disarticulation. Labium indicated by black arrow. b) Larva in ventral attitude with labial mask completely disarticulated. Labium indicated by black arrow. c,d) Larva in dorsal attitude with labium completely disarticulated. In image ‘d’, the specimen is also in late state of decomposition evidenced by the transparent cuticle. Labium indicated by black arrows. e) Four larval carcasses with legs and labial masks disarticulated. Labium indicated by black arrows, and fragments of legs (mainly hind) indicated by red arrows.

Regarding the disarticulation observations, the first element to disarticulate in the larvae was the labium. Almost half of all the specimens analyzed (n = 93) had their labium disarticulated at the end of the experiments (Figs 10 and 11; S2 Table), and it tended to disarticulate within one week after death in the majority of the specimens (n = 62; Figs 10 and 11). We considered the labium disarticulated when it was distended enough to observe its tip (i.e., the prementum) on dorsal view (Fig 11c–11e). Regarding only the specimens from the flotation tests, 46% of the carcasses of the freshwater group (0.5‰) showed labium disarticulation (n = 11), 26% of the saltwater group (35‰, n = 5), and only 10% of the carcasses in hypersaline water (80‰, n = 2). The G-test for labium disarticulation showed a significant difference for different salinity concentrations (p-value = 0.008, df = 3). The G-test for the labium mask condition showed a significant difference between the four experimental groups (p-value < 0.05, df = 3).

Among anisopteran groups (n = 233), besides the labium, the legs were also the first to disarticulate, especially under mechanical disturbance (25% of specimens had at least one leg missing at the end of disarticulation observations, n = 59) (Fig 11e), and the disarticulation of the abdomen from the thorax was rare, occurring in only three specimens. Regarding the three analyzed damselflies larvae (Zygoptera), the caudal gills were the most easily disarticulated elements, with all specimens losing them. Raw data on the experiments with Odonata are available in S2 Table.

### Biostratinomic data of fossil mayflies

**Fig 12:**
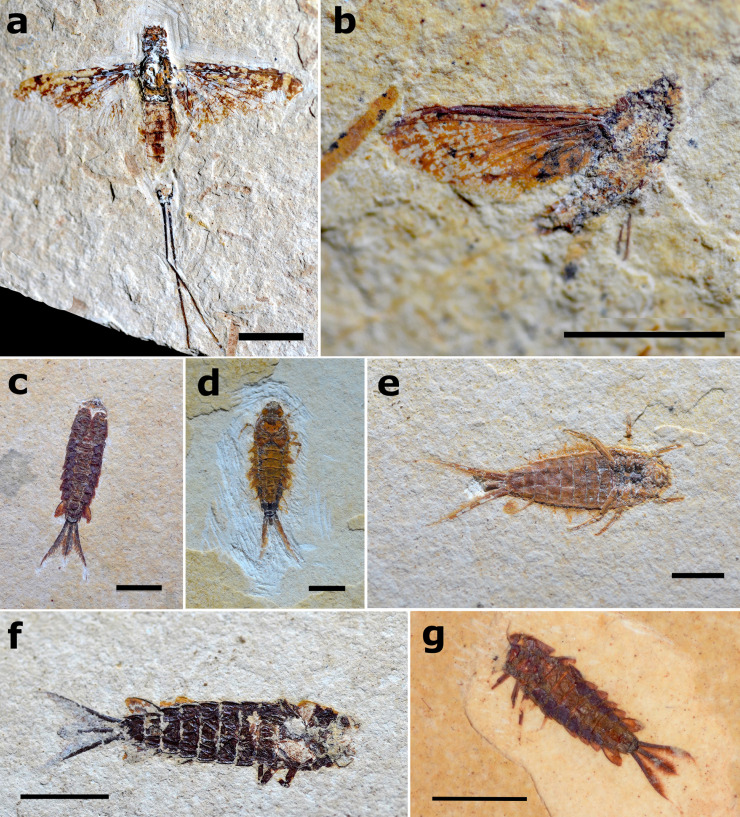
Fossils of Hexagenitidae from the Crato Formation. a) Winged specimen, MPSC I 4287, preserved in dorsal attitude with wings open and spread out. Scale 5 mm; b) Winged specimen, MPSC I 409, preserved in lateral attitude with wings closed. Scale 5 mm; c) Larval specimen, MPSC I 5229, preserved in dorsal attitude. Scale 3 mm; d) Larval specimen, LPU 1698, preserved in dorsal attitude. Scale 3 mm; e) Larval specimen, MPSC I 5230, preserved in ventral attitude and displaying the typical death position recovered in extant larvae. Scale 3 mm; f) Larval specimen, LPU 1697, preserved in ventral attitude. Scale 4 mm; g) Larval specimen, MPSC I 5227, preserved dorsolateral attitude. Scale 5 mm.

Most winged Hexagenitidae preserved in the Crato Formation limestones are preserved either in dorsal or ventral view, and have their wings open and spread out (approximately 70% of specimens, n = 22), while approximately 25% of specimens (n = 8) are in lateral position and have their wings adducted, and only one specimen was preserved as mere isolated wings (S3 Table). Among larval Hexagenitidae from the same unit, most are preserved in dorsal view (61% of the specimens, n = 140), followed by ventral view (22% of specimens, n = 50), and lateral view (10% of specimens, n = 24) (Fig 12). The remaining specimens have a non-discernible view due to poor preservation (n = 16). A total of 23% of the Hexagenitidae larval specimens of the Crato Formation (n = 53) preserved in dorsal or ventral view are similar to the death position we observed in the larvae of *C. capixaba* (in dorsal view, legs not flexed but extended laterally near the body, with the anterior legs extended forward; and in ventral view, legs flexed over the body) (Fig 12; S3 Table).

**Fig 13:**
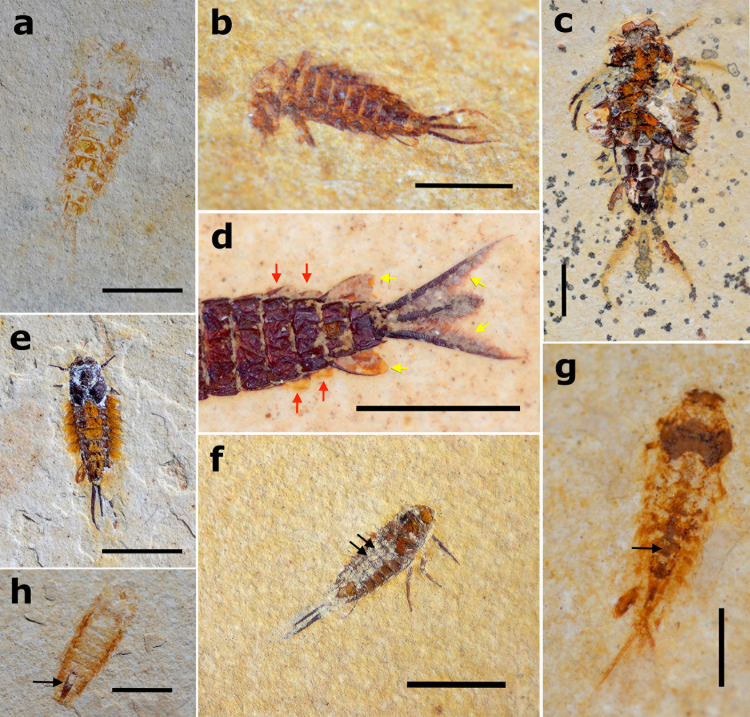
Hexagenitidae larval specimens in different disarticulation degrees. a) Unnumbered-121 LPU specimen with disarticulated thorax. Scale bar 3 mm; b) Specimen MPSC I 293 with thorax about to disarticulate. Scale bar 5 mm; c) Specimen GP/1T 2583, an example of a specimen considered completely articulated in our study – even if the preservation is hampered by weathering, for instance. Scale bar 3 mm; d) Part of specimen LPU 1697 evidencing its well-preserved details such as the abdominal cuticle texture, gills V–VI closed (indicated by red arrows) and VII (indicated by black arrow), and setae on caudal filaments (indicated by yellow arrows). Scale bar 5 mm; e) Another example of unnumbered-53 LPU specimen considered completely articulated in this study. Scale bar 5 mm; f) Specimen MPSC I 5224, considered completely articulated in this study and preserving micro textures on its dorsal cuticle (indicated by black arrows). Scale bar 5 mm; g) Unnumbered-12 LPU specimen showing signs of cuticle denaturation but still articulated; digestive tube well visible in its all length (indicated by black arrow). Scale bar 5 mm; h) Unnumbered-35 LPU specimen showing signs of cuticle denaturation, remaining only its lower digestive tube preserved (indicated by black arrow); caudal filaments, gills, and thorax disarticulated. Scale bar 3 mm.

Hexagenitidae are often completely articulated (81% of all specimens, n = 214, including larvae and winged forms). Less than 5% of larval specimens (n = 13) have a disarticulated thorax (Fig 13a and 13b). Several specimens have fragile structures preserved, like setae on caudal filaments (31% of specimens, n = 71) (Fig 13c–13f). Also, a small percentage of Hexagenitidae larvae (14%, n = 34) have no cuticle preserved and show their inner digestive tube (Fig 13a, 13g, and 13h; S3 Table).

**Fig 14:**
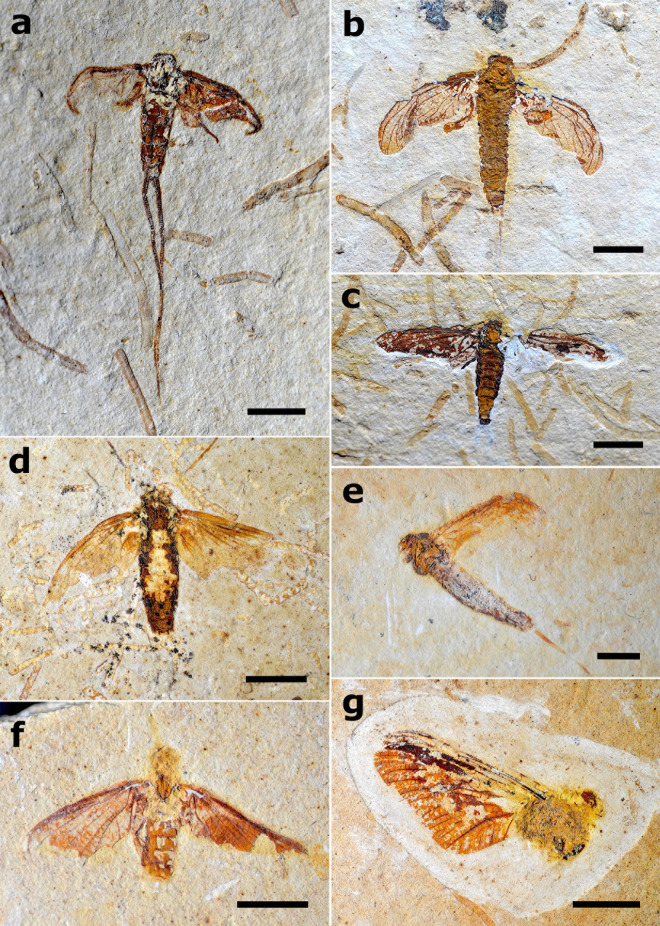
Hexagenitidae winged specimens in different disarticulation degrees. a) Specimen MPSC I 4286 with four legs missing and wings withered. Scale bar 4 mm; b) Unnumbered LPU specimen with all legs and part of caudal filaments missing; wings are also partially withered. Scale bar 4 mm; c) Specimen MPSC I 4422 with five legs, caudal filaments, and right hind wing absent, as well as border of forewings. Scale bar 4 mm; d) Specimen GP/1E 9562 with four legs, caudal filaments, and border of forewings absent. Scale bar 4 mm; e) Specimen GP/1E 7221 with all legs (except one pair of coxae) absent and with wings withered. Scale bar 3 mm; f) Specimen GP/1E 6763 with all legs and end of abdominal segments missing; the borders of all wings are also absent. Scale bar 5 mm; g) Specimen GP/1E 9034 with all legs, hind wings, and abdomen missing. Scale bar 5 mm.

In alate forms, the legs are the body part most likely to be absent (i.e., more easily subject to disarticulation), with at least four legs absent in 90% of specimens (n = 28) with body parts absent (four legs absent in 4 specimens, five legs absent in 7 specimens, and all legs absent in 17 specimens), followed by the caudal filaments, with at least one of them being absent in 42% of the analyzed specimens (n = 13). In 25% of the specimens (n = 8), there is at least one disarticulated segment in the abdomen. The less frequently absent body elements are the hind wings (absent in one specimen), the whole body (head, thorax, and abdomen combined, remaining only isolated wings, absent in one specimen), and one of the forewings (absent in one specimen) (S3 Table). Also, 18% of winged fossils (n = 7) present their wings withered in different degrees, similar to the dehydrated and decomposed state observed during our actualistic experiments (see above) (Fig 14).

**Fig 15:**
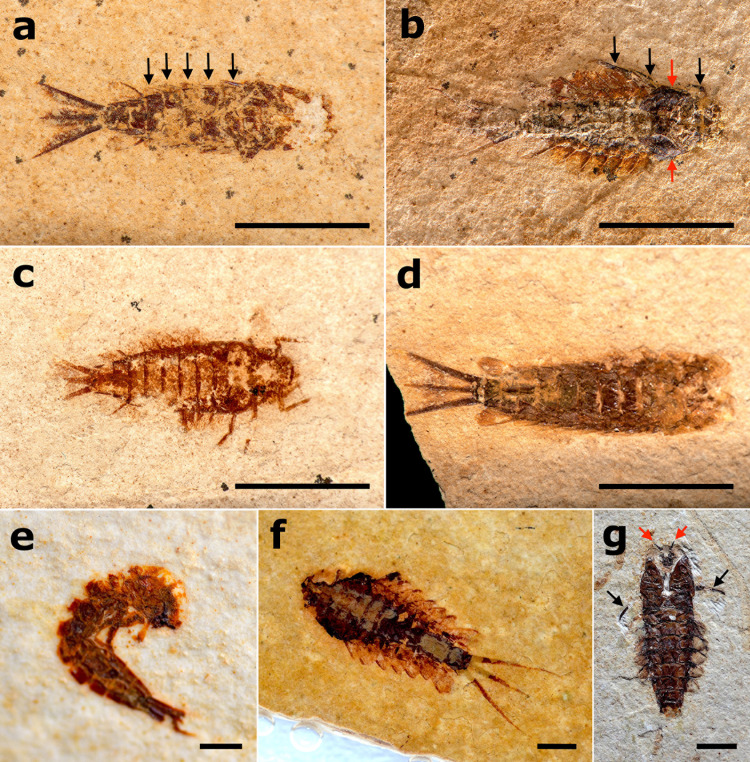
Hexagenitidae larval specimens in different disarticulation degrees. a) Specimen MPSC I 2507 in ventral attitude with antennae missing; gills II-VI preserved close to the body and left ones (indicated by black arrows). Scale bar 5 mm; b) Specimen MPSC I 2533 in dorsal attitude. Three legs are partially preserved (indicated by black arrows), as well as all their gills in yellowish coloration (except gill VII), wing pads are also preserved and indicated by red arrows. Scale bar 5 mm; c) Specimen MPSC I 2509 preserved in ventral attitude; five legs are almost completely preserved in a natural postmortem position. Scale bar 5 mm; d) Specimen MPSC I 2516 preserved in ventral attitude with all legs missing. Scale bar 5 mm; e) Specimen MPSC I 1336 was preserved with the abdomen about to disarticulate in curved lateral attitude; part of caudal filaments is also missing. Scale bar 2 mm; f) Unnumbered LPU specimen preserved in dorsal attitude, evidencing all gills preserved and all legs missing. Scale bar 2 mm; g) Unnumbered LPU specimen preserved in dorsal attitude; two legs are partially preserved (indicated by black arrows) as well as antennae (indicated by red arrows) and wing pads, while part of its gills and caudal filaments were disarticulated. Scale bar 3 mm.

In the larvae, the legs are the body part more frequently absent, with at least one leg missing in 88% (n = 202) of specimens lacking any body parts (missing one leg in 7%, n = 16; two legs in 5%, n = 11; three legs in 12%, n = 28; four legs in 9%, n = 22; five legs in 6%, n = 13; and all legs missing in 50%, n = 115). They are followed by the antennae (absent in 64% of the specimens, n = 147) and at least two gills (absent in 37% of the specimens, n = 85). Finally, at least parts of the caudal filaments are absent in 34% (n = 78) of the incomplete specimens (parts of caudal filaments missing in 5%, n = 11; paracercus absent in 6%, n = 13; one cercus absent in 2%, n = 5; cerci absent in 3%, n = 6; and all the caudal filaments absent in 19%, n = 43). The less frequently absent body elements among the larvae are abdominal segments (absent in only one specimen), cuticle (absent in two specimens), the head (absent in 4% of specimens, n = 12), and the head and thorax combined (absent in 5% of the specimens, n = 13) (Fig 15). We also found that 15% of the specimens (n = 34) are preserved with signs of cuticle denaturation in different degrees, made evident by the recognizable digestive tube due to cuticle tissue loss (Figs 13g–13h; 14; S3 Table).

### Biostratinomic data of fossil dragonflies

**Fig 16:**
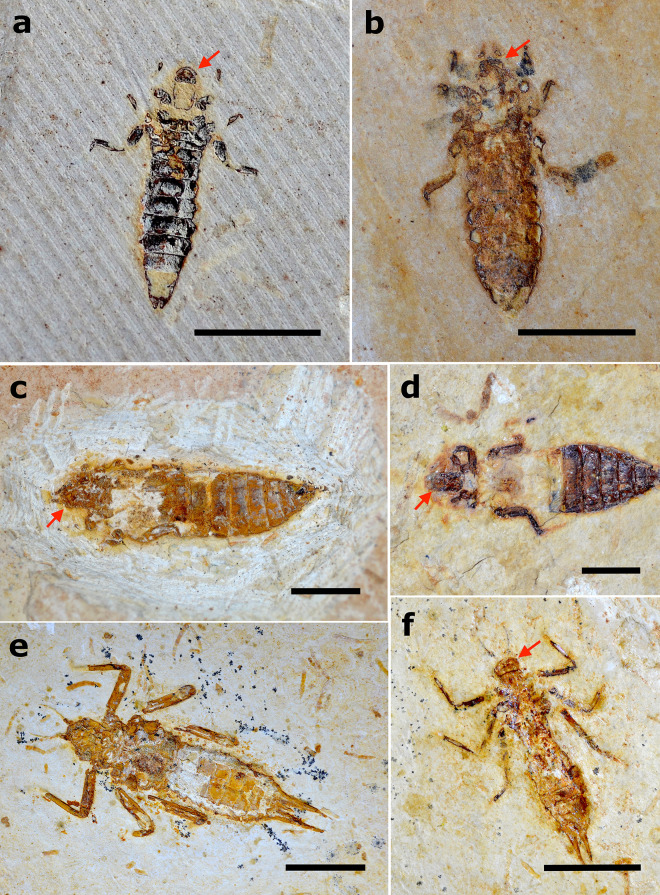
Dragonfly larval specimens with labial masks articulated in different degrees. a) Proterogomphidae specimen LPU 1102 in ventral attitude evidencing labial mask totally disarticulated/distended (indicated by red arrow); specimen was also preserved in a natural death position. Scale bar 5 mm; b,c) Proterogomphidae specimens MPSC I 388 and GP/1E 6891 in ventral attitude showing labial mask still articulated or only partially disarticulated, evidenced by the visualization of antennae in both specimens. Scale bar 5 mm; d) Proterogomphidae specimen MPSC I 384 in ventral attitude evidencing disarticulated labial mask (indicated by red arrow). Scale bar 5 mm; e) Nothomacromiidae specimen MPSC I 709 in dorsal attitude. Scale bar 10 mm; f) Nothomacromiidae specimen in ventral attitude with labial mask still articulated (indicated by red arrow). Scale bar 10 mm.

**Fig 17:**
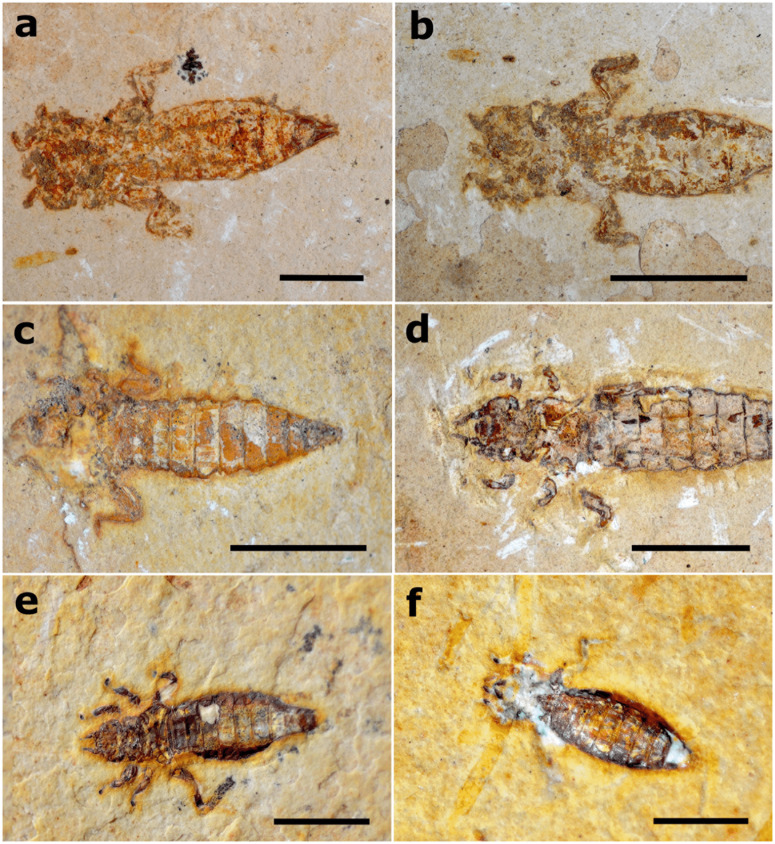
Proterogomphidae fossils displaying natural postmortem position. a) Specimen GP/1E 9220; b) Specimen GP/1E 6807; c) Specimen GP/1E 6806; d) Specimen GP/1E 8699. The natural death position was slightly modified in this specimen since the right hind leg is close to the body and not spread open as the left leg; e) Specimen MPSC I 389. Hind legs showing signs of minor transport since the right one is disarticulated at posterior segments, and the left is about to disarticulate; f) Specimen MPSC I 1355. Scale bars 5 mm.

Fossil dragonfly larvae from the Crato Formation are often completely articulated (64% of specimens, n = 29). Similarly to the actualistic experiments with anisopterans, the analyzed fossils never displayed thorax, head, or abdomen disarticulated since those are extremely robustly attached structures in dragonflies, differing from the damselflies’ larvae (Figs 16 and 17; S4 Table).

In contrast to the observed actualistic results with extant specimens, we rarely found disarticulated labial masks in the fossils from the Crato Formation, being disarticulated in only 2 specimens (Fig 16). Concerning the other corporal elements, 31% of the fossils (n = 14) have at least the final segments of the legs missing (3 specimens lacking only the tarsi of some legs, 4 specimens missing one leg, 2 lacking two legs, 3 missing three legs, 1 lacking four legs, and 1 missing five legs), and 17% of specimens (n = 4) have their antennae absent. Forty-seven% of the analyzed dragonfly larvae (n = 21) from the Crato Formation display the same natural postmortem position that we observed in the extant larvae, with the delicate hind legs still preserved, although in five cases at least one segment from the hind leg disarticulated (Figs 16a, 16b and 17; S4 Table). Although marginally non-significant (p-value = 0.050, df = 4), the G-test results showed a tendency for gomphid larvae to display a natural post-mortem position, with the hind legs extended laterally and the forelegs extended close to the body.

## Discussion

Proxies used for determining if assemblages suffered transport to the depositional site usually involve the study of abundance, the degree of tissue breakage, disarticulation and fragmentation of individuals, and the attitude of fossils in sediments, although disarticulation can occur naturally over time [35,62]. During our experiments, the thorax was the most likely element to disarticulate first in *C. capixaba* larvae (even more easily in their exuvia), especially in situations of denaturation acceleration, such as in warmer water. As the Hexagenitidae larvae from the Crato Formation rarely present a disarticulated thorax, this indicates that their carcasses suffered little or no disturbance and that the decomposition process must also have been slow. Additionally, rapid mineralization could be another important factor [62]. This also could suggest that the carcasses suffered minimal transport, corroborating that the group was living in the depositional site (i.e., were autochthonous), as pointed out by [21].

Another set of biostratinomic data additionally supports the idea that Hexagenitidae carcasses suffered little or no disturbance and/or slower decomposition. All extant mayflies that died in the experiments under higher temperatures presented straighter bodies (i.e., not curving), which could be related to the acceleration of cuticle denaturation at high temperatures [63]. But in the fossil record, the few larvae that are preserved laterally display a natural curvature in their body. Only a few fossil Hexagenitidae larvae were recovered without cuticle preserved and showing their inner digestive tube. Here, we interpret them as larvae that underwent enough decomposition to lose their cuticle before undergoing diagenesis, similar to the experiments in which the specimens went through cuticle denaturation after many days (when disarticulation tests ceased). Finally, fossil specimens lacking their wings’ margins and/or with their wings withered, which could indicate longer decay time or even disturbance of the carcass, are rare in the Crato fossil record. However, these latter could also simply represent subimagoes that did not complete the full opening of wings after emergence, since some degree of withering of the wings is common in this alate stage [40].

All the Odonata groups recovered in the Crato Formation have been presumed in the past as allochthonous to the depositional site [13,18,5]. However, we assume otherwise for Proterogomphidae and Nothomacromiidae, which are the groups of dragonflies with the majority of larval representatives preserved in the Crato Formation [18]. During our experiments, we observed that once dead, the dragonflies stayed in a characteristic position with the hind legs distended laterally and the forelegs extended anteriorly. However, as soon as any mechanical disturbance occurs, the segments of the hind legs easily disarticulate. Seventeen of the 28 analyzed proterogomphid larvae from the Crato Formation also presented this pre-disturbance pose (Fig 17), and 14 of them still had all segments of the delicate hind legs attached, indicating minimal disturbance of the carcasses after death (e.g., transport). Moreover, since we rarely found the labial mask preserved as visibly disarticulated, we could at first hypothesize that this structure disarticulates so early that it is the first part of the body to completely detach, thus being absent from the fossil record. However, it is possible to see the structure still attached, but not distended or disarticulated, in at least 14 of the analyzed fossils. Also, in none of our experiments did the strongly adhered labium detach from the body, as in the case of legs. These observations indicate an autochthonous state for those groups. Although Proterogomphidae and Nothomacromiidae larvae are not as commonly found in the Crato Formation limestones as Hexagenitidae larvae [21,64] – for instance, only two specimens in total were recovered in the controlled collection, they could be more abundant in different strata than those excavated at the Antonio Minelon Mine, pertained to smaller assemblages, or were more common at a different part of the palaeolake.

In our experiments, all dragonfly carcasses immediately floated under hypersaline conditions. Carcasses immersed in hypersaline conditions had slower decomposition and disarticulation times, while the carcasses immersed in freshwater or lower salinity levels took days to float and had accelerated decomposition when compared to carcasses in hypersaline water. This late buoyancy, which started in the abdomen, was probably due to the time taken to accumulate decay gases. Salinity levels, therefore, must play a key role in increasing the buoyancy of the carcasses and thus their transport, besides increasing the water’s surface tension [65], but also improving the preservation of carcasses. Finally, we also observed that the angle of the legs of dragonflies’ carcasses was not affected by hypersaline conditions, which probably would only happen if the individuals were still alive (similarly to results of [38] with spiders), and therefore the legs’ angle can only be a useful proxy for individuals that reached the depositional site alive and not as transported carcasses. Thus, our results suggest that, although some authors have interpreted the Odonata larvae as allochthonous to the Crato Formation depositional sites [13,5], at least the Proterogomphidae and Nothomacromiidae individuals probably lived close and were not transported for long distances (i.e., were autochthonous to parautochthonous).

When considering certain temporal phases of and/or certain strata of the water body of the Crato Formation, there is a good deal of hypotheses in favor of a more brackish or saline palaeolake environment [66,67,68,69,70,71,41,72,73,26,21] and some that support a more lacustrine water setting [74,28,4]. In our experiments, specimens allocated at lower salinity levels defecated in unusually large amounts long before death. These larvae were probably releasing a lot of salt directly into their feces since, in insects, the excretory system is linked to the digestive system by the Malpighian tubules [75]. Unfortunately, we have not seen anything that could be related to this process in the Crato mayflies.

Our experiments have shown that when small insects such as mayflies die in sub-aerial conditions and are transported to the water, there are few possibilities of overcoming the surface tension and sinking. In mayflies, the subimagoes present wings covered with microtrichia that could keep them afloat, and males potentially could float more easily since their abdomen is filled with air, instead of eggs [40]. Our results agree with actualistic data from Martínez-Delclòs and Martinell [35], who studied different terrestrial insects and concluded that it is difficult for small insects to sink. Their sinking could be generated by some disturbance (e.g., strong water currents), which cannot be completely discarded for the fossils we analyzed, but the Crato Formation’s limestones are generally inferred as a calm depositional site due to their fine-scale parallel lamination [76]. Thus, another phenomenon probably occurred during carcass sinking, such as the formation of floating microbial mats or biofilms, which, when lumped around a carcass, make it denser, breaking the water tension and making it descend (see Fig 8). This adds to the hypothesis that the formation of benthonic microbial mats, directly on the bottom of the depositional site (after sinking), improves the preservation of carcasses [70,77,26,28,31]. We hypothesize that, additionally, entombment by microbial biofilms while floating was also important to improve the conservation of articulated insect fossils in the Crato Formation, as already acknowledged as important for the preservation of insects of the Florissant Fossil Beds, United States [78,79], and in the Crato Formation itself by Dias and Carvalho [50], who suggested this hypothesis while studying fossil crickets. They stated that “The entry of water into the tracheal breathing system in association with carcass infestation by microbial communities increases the density of the remains and enables them to reach the palaeolake substrate faster. Therefore, insect carcasses are trapped and enveloped by microbial communities from their initial fall into the water body to the substrate”. Consequently, our study confirms, experimentally and for other taxa, what was theoretically suggested for crickets by Dias and Carvalho [80].

The degradation of chitin is catalyzed by chitinases, which are found in organisms such as fungi and bacteria [81]. Insect cuticles can be decomposed by bacteria from the genus *Chitinophaga* (or chitinolytic bacteria), which are almost exclusively aerobic, found in a wide range of habitats, and can form superficial biofilms [35]. *Streptomyces* species, which have been thoroughly studied, decompose solid chitin pieces rapidly, in large part because of their ability to penetrate substrates (or water) in association with fungal hyphae, such as those of *Saprolegnia* sp. [82,83]. The microbial growth we saw taking over the studied carcasses had definitely fungi in its composition, given its hyphae-like morphology. Similar bacteria/fungi could potentially decompose carcasses in the Crato palaeolake while, at the same time, acting as an anchor, because they increase density and thus help the insect to overcome the surface tension and sink to the bottom. They also act as a type of cohesion mechanism for the remains, hindering fragmentation and disarticulation [79]. However, cuticular decomposition by chitinophagous consortia is greatly hindered by anoxia and low temperatures, so the decomposition could stop or be delayed as soon as the carcass reaches the anoxic bottom of the Crato Formation’s depositional site.

Although their experimental setup differs from ours, Martínez-Delclòs and Martinell [35] observed in laboratory conditions that, without biological or physical disturbing agents, when reaching the bottom, insects could remain there for one year without becoming disarticulated, but over time disarticulation occurs around the joint zones due to the natural decomposition of carcasses [35]. This differed from our experiments, in which this process lasted roughly only a month. We also observed that the more delicate the body, the faster this occurred; for instance, it was faster in small-sized mayfly larvae than in the more robust dragonfly larvae.

## Conclusions

Our biostratinomic data and actualistic approach help to explain the fossilization process and the past environmental conditions the aquatic insects from the Crato Formation went through. Our results corroborate the mayfly group Hexagenitidae (specifically *Protoligoneuria limai* assemblages) as autochthonous to the depositional site of the Crato Formation, a condition that was probably only shared with small populations of proterogomphids, and seasonally with *Dastilbe* juvenile fish [21]. *Protoligoneuria limai* is the most abundant invertebrate taxon of the Crato Formation, presenting complete ontogenetic series, and has already been reported in mass mortality events [21]. Only 4% of hexagenitid larvae we analyzed had a disarticulated thorax, which also agrees with autochthony. Based on our experimental evidence, we conclude that, at least for the studied mayfly larvae, the high completeness rate would not have been possible if the carcasses had been transported from outside the depositional site.

Our observations are also consistent with Proterogomphidae and Nothomacromiidae dragonflies living close to the depositional site and not being transported for long distances, as evidenced by the completeness of their fossils. We assume this due to the rare examples of disarticulated labial masks in the fossils, which is further evidence of non-allochthony. Also, several fossils we analyzed had their body preserved in a natural death pose, in some cases including all segments of the leg preserved (extremely delicate elements that are disarticulated by minimal currents or mechanical friction). However, a thorough review of dragonflies from the Crato Formation is crucial for further taphonomic elucidations, and future interpretations should be based, preferably, on specimens collected under controlled excavations to remove collection bias that privileges well-preserved specimens, a common problem when studying Crato fossils [84,85]. The taphonomy of insects from the Crato Formation will be better understood only when we also study the poorly preserved fossils.

One may also hypothesize some details on the palaeoenvironment of the Crato Formation based on these comparisons between its aquatic insects’ fossils and experimental actualistic data. The palaeolake complex of the Crato Formation [4], in which these autochthonous populations were living, had most probably floating microbial biofilms in its surface waters, instead of only on the bottom (as suggested by a variety of works, such as [70,77,26,28,31]), similarly to the ones that grew in extant carcasses during our experiments of disarticulation, helping the carcasses to sink and keeping their body parts cohesive. Floating microbial biofilms are therefore a possibility, but other experiments, and microscopic and chemical analyses are needed to better support it.

## Supporting information

S1 Table Raw data from experiments with extant Ephemeroptera specimens. (XLSX)

S2 Table Raw data from experiments with extant Odonata specimens. (XLSX)

S3 Table Raw data from biostratinomic analysis of fossil Ephemeroptera. (XLSX)

S4 Table Raw data from biostratinomic analysis of fossil Odonata. (XLSX)
